# Strategies to Use Nanoparticles to Generate CD4 and CD8 Regulatory T Cells for the Treatment of SLE and Other Autoimmune Diseases

**DOI:** 10.3389/fimmu.2021.681062

**Published:** 2021-06-15

**Authors:** David A. Horwitz, Sean Bickerton, Antonio La Cava

**Affiliations:** ^1^ General Nanotherapeutics, LLC, Santa Monica, CA, United States; ^2^ Department of Medicine, Keck School of Medicine, University of Southern California, Los Angeles, CA, United States; ^3^ Department of Biomedical Engineering, Yale University, New Haven, CT, United States; ^4^ Department of Medicine, University of California, Los Angeles, Los Angeles, CA, United States

**Keywords:** nanoparticles, regulatory T cells, systemic lupus erythematosus, autoimmunity, treatment, antigen-presenting cell, dendritic cell

## Abstract

Autoimmune diseases are disorders of immune regulation where the mechanisms responsible for self-tolerance break down and pathologic T cells overcome the protective effects of T regulatory cells (Tregs) that normally control them. The result can be the initiation of chronic inflammatory diseases. Systemic lupus erythematosus (SLE) and other autoimmune diseases are generally treated with pharmacologic or biological agents that have broad suppressive effects. These agents can halt disease progression, yet rarely cure while carrying serious adverse side effects. Recently, nanoparticles have been engineered to correct homeostatic regulatory defects and regenerate therapeutic antigen-specific Tregs. Some approaches have used nanoparticles targeted to antigen presenting cells to switch their support from pathogenic T cells to protective Tregs. Others have used nanoparticles targeted directly to T cells for the induction and expansion of CD4+ and CD8+ Tregs. Some of these T cell targeted nanoparticles have been formulated to act as tolerogenic artificial antigen presenting cells. This article discusses the properties of these various nanoparticle formulations and the strategies to use them in the treatment of autoimmune diseases. The restoration and maintenance of Treg predominance over effector cells should promote long-term autoimmune disease remission and ultimately prevent them in susceptible individuals.

## Introduction

A major unmet need in chronic immune-mediated inflammatory diseases that include autoimmune diseases, graft versus host disease and allograft graft rejection is to achieve long-term remission. Most current approaches use agents that are only partially effective because they not only suppress pathologic cells but also the cells that are required to control those pathologic cells. Moreover, the broad immunosuppressive effects of pharmacological and/or biological agents are often accompanied by toxic side effects. Fortunately, novel strategies with more selective cellular targets (and thus more effective and less toxic) are being developed.

Autoimmune diseases are generally T cell-dependent disorders of the immune regulation. The immune system is constitutively highly active with a rapid turnover of T regulatory cells (Tregs) and antigen-presenting dendritic cells (DCs). Homeostatic regulatory mechanisms control immune cells with dual functions: 1) they fight infectious agents and 2) also prevent the emergence of potentially pathologic self-reactive cells not eliminated at birth. In health, regulatory populations of CD4+ and CD8+ Tregs keep these cells dormant, and interactions between tolerogenic DCs and Tregs maintain immune tolerance. In autoimmune diseases, instead, homeostasis becomes dysregulated and immunogenic DCs enable pathogenic T effector cells to predominate over the Tregs (1). A prototypical disorder of immune regulation is systemic lupus erythematosus (SLE), a multisystem autoimmune disease (2). In SLE both CD4+ and CD8+ Treg function is decreased (3).

Several therapeutic approaches have been developed to restore normal numbers and/or function of Tregs when abnormal. One approach that has reached clinical trials has been to isolate and expand the small numbers of Tregs present in the peripheral blood. The adoptive transfer of expanded autologous CD4 Tregs has been used to treat various autoimmune diseases, graft versus host disease and to prevent solid organ graft rejection (4). Adoptive CD4+ T cell therapy in one case of lupus with skin disease revealed evidence of T reg activation (5). Although the adoptive transfer of expanded polyclonal CD4 Tregs appears to be safe, the cost and technical complexity to expand autologous Tregs have limited this approach (6). An alternative strategy has been the induction/expansion of Tregs *ex vivo*. The cytokines interleukin (IL)-2 and transforming factor-beta (TGF-β) are essential for the generation, function and survival of CD4 Tregs (7, 8). In SLE, the production of IL-2 and TGF-β is decreased (9, 10). To treat SLE and other autoimmune diseases with low IL-2 production, one could induce and expand autologous SLE CD4 Tregs *ex vivo* with IL-2 and TGF-β for subsequent adoptive transfer of these cells back to the donor (11). Although this Treg-based therapeutic approach has been successful in mouse models, it has not yet reached the clinic. The possibility to induce and expand *in vivo* Tregs has recently been considered through the use of nanoparticles (NPs). Formulated NPs with the potential to reset the homeostatic mechanisms restoring Treg predominance are discussed here. Since DCs control T cell differentiation, one approach is to switch disease-associated immunogenic DCs to tolerogenic DCs (which induce and expand Tregs). Another approach directly targets T cells and increases functional CD4+ and CD8+ Tregs. We discuss how the immunotherapeutic use of NPs could lead to the reversal, long-term remission, and ultimately, prevention of autoimmune diseases.

## Nanoparticles in Immunotherapy

Nanoparticles engineered to target specific cells or tissues with a high drug loading capacity represent a new generation of drug delivery systems for many biomedical indications. Nanoparticles are constructed using natural or synthetic materials with well-established safety record and have a typical diameter ranging from 0.1 to 1000x10^-9^ m (1 nanometer, which is 10x the size of an atom). The motivation for using such systems derive from the fact that viruses and pathogens that elicit or subvert immune responses are, in essence, small particles endowed with the ability to interact with - or avoid - immune cells in a variety of ways. Nanoparticles currently used consist of both organic or colloidal NPs that can be taken up by cells of the reticuloendothelial system. These include the phagocytic cells of the innate immune system such as macrophages, DCs and neutrophils. Other NPs can be surface-modified to target specific lymphocyte populations.

**Figure 1:**
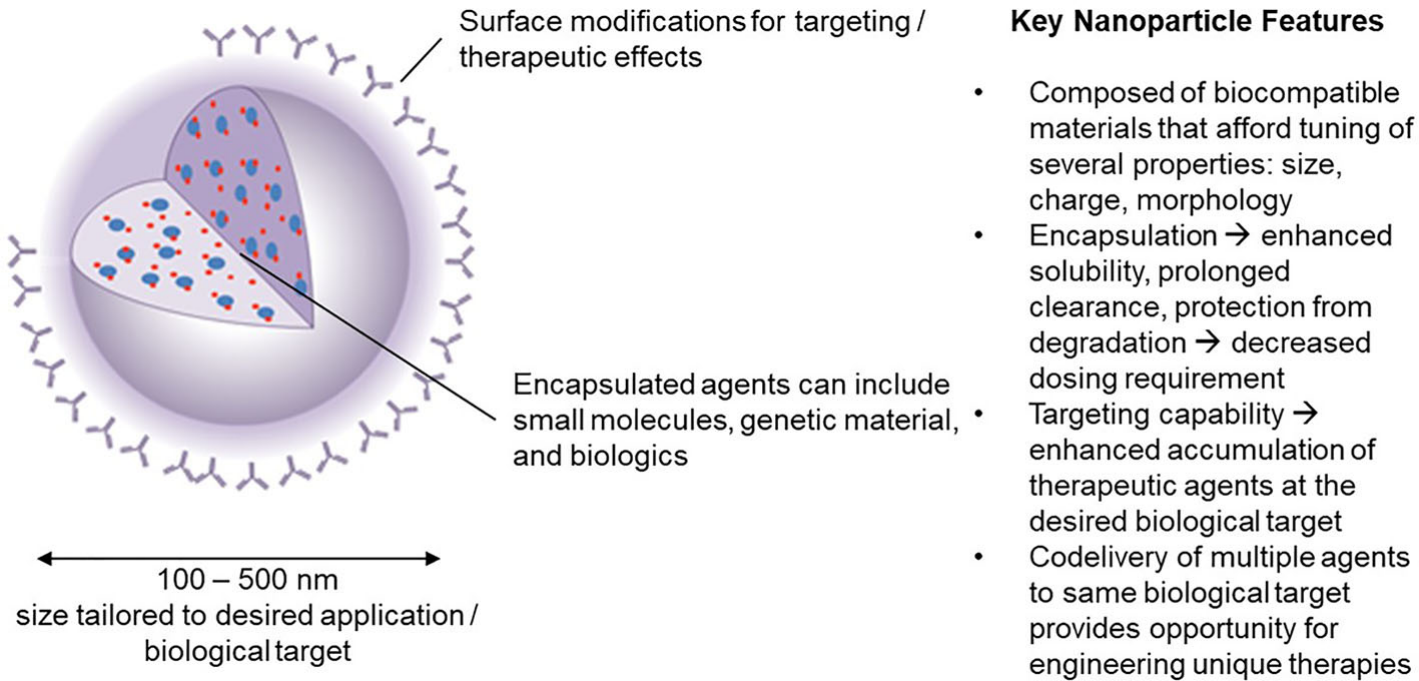
Nanoparticle carriers offer a unique set of characteristics that have inspired significant interest in their use in engineering novel immunotherapies in the field of tolerance induction.

Advantages of NPs over traditional drugs include: 1) markedly decrease the amount of a biological agent delivered by 100 to 1000-fold when targeted to specific cells (by increasing the local concentration following release). This reduces the side effects as well as the cost. 2) improve the delivery of insoluble drugs and maximize bioavailability; 3) combine therapeutic agents with a diagnostic, resulting in “theranostic” agents. The durability of the concept is an indication of its appeal in developing immunomodulatory strategy technologies. The potential to assemble such materials on nanoscale dimension facilitates circulation in the blood, biodistribution to lymph nodes, interaction with extracellular receptors (if targeted appropriately) and intracellular accumulation without compromising normal physiologic functions. We focus here on the application of nanoparticles in the size range 100-500 nm (**Figure 1**).

Nanoparticles are currently being tested for the treatment of autoimmune disease because they can be engineered for three distinct uses: 1) they can function as carriers of biologic agents and small molecule drugs, 2) they can be anti-inflammatory, or 3) tolerogenic (12, 13). Taking advantage of the fact that they are phagocytosed by macrophages, NPs can encapsulate agents that polarize those cells to become anti-inflammatory. These agents include cytokines such as IL-10, statins, angiotensin receptor blockers, or peroxisome proliferator-activated receptor-γ (PPARγ) agonists (14). Nanoparticles loaded with biological agents such as tumor necrosis factor antagonists ameliorate inflammatory arthritis (15). Here we concentrate on the use of NPs to induce and expand Tregs.

The effects of NPs are determined by their size, biodistribution and route of administration. Particles smaller than 6 nm drain to the blood while particles larger than 9 nm drain preferentially to lymphatics. Particles 20 to 100 nm are taken up by liver sinusoidal cells or macrophages. Particles 100 to 200 nm traffic to the spleen and liver, and those up to 5 µm will accumulate in the spleen. NPs delivered by intravenous injection target APCs in the spleen and liver. Those delivered by subcutaneous injection are preferentially taken up by DCs in draining lymph nodes.

The materials used for the preparation of NPs can include metals, liposomes and synthetic and natural polymers (16–19). Specifically, polymers fabricated from polylactides (PLA) and copolymers of lactide and glycolide (poly-lactic-*co*-glycolic acid, PLGA) have established commercial use in humans and have a long safety record (20, 21). These systems have several features that make them ideal materials for the fabrication of anti-inflammatory or tolerogenic nanosystems: 1) control over the size range of fabrication, down to 100 nm and potentially even lower (an important feature for passing through biological barriers); 2) reproducible biodegradability without the addition of enzymes or cofactors; 3) capability for sustained release of encapsulated, protected cytokines or other agents that may be tuned in the range of days to months by varying factors such as the PLA to polymers of glycolic acid (PGA) copolymer ratios, potentially abrogating repetitive administrations, 4) well-understood fabrication methodologies that offer flexibility over the range of parameters that can be used for fabrication, including choices over the polymer material, solvent, stabilizer, and scale of production and 5) control over surface properties facilitating the introduction of targeting ligands on the surface (18, 22).

While other materials can be considered such as metal oxide NPs - which can be conjugated with antigens, targeting ligands and immunomodulators on the cell surface - these do not facilitate sustained release and are limited to applications that do require biodegradability. Renal clearance is the major clearance pathway with such systems and requires them to be ultra-small (<50 nm). Given the potential safety issues with long-term use, liposomes that carry antigen, NF-kB inhibitors, or immunosuppressive drugs are often safer options and do not require stringent size engineering criteria. They have been used to suppress arthritis and lupus (23, 24) and variants of liposomes with a hydrogel interior (to facilitate sustained release) have been developed and utilized for the delivery of biologics and small molecule drugs in lupus therapy (24).

The appeal of biodegradability of NPs for controlled release of encapsulant together with safety requirements have led to the wide use of synthetic biopolymers as materials for construction of biodegradable NPs. The most widely used NPs are synthetic polymers, such as PLA or PLGA. Unlike liposomes, which burst release unless lipids are cross-linked or the interior is modified with a hydrogel (12), these solid biodegradable polymer particles are stable over time in aqueous media, releasing encapsulant slowly and, in addition, they can be manufactured by a number of methodologies and facilitate encapsulation of hydrophobic moieties such as rapamycin, mycophenolic acid, vitamin D3 and dexamethasone (25) through an entanglement with the hydrophobic polymer core (24, 26–28). One group compared the tolerogenic effects of PLGA NPs with TMC-TPP (N-trimethyl chitosan tripolyphosphate) NPs. They found that PLGA NPs enhanced production of retinal dehydrogenase by APCs. This enzyme increases retinoic acid which enhanced CD4+Foxp3+ Tregs induced by TGF-β (29). Clinically, this is of interest because IL-2 and TGF-β induce human naïve CD4 cells to express FoxP3 but, unlike mice, these cells lack strong suppressive effects. Adding all-trans retinoic acid to IL-2 and TGF-β markedly increased the protective properties of the Tregs to levels equivalent to mouse Tregs (30). PLGA NPs also increase the stability of induced CD4 Tregs. As will be discussed below, mouse CD4+ cells induced to become CD25+Foxp3+ Tregs with IL-2- and TGF-β-loaded PLGA NPs were more stable than Tregs induced with soluble IL-2 and TGF-β (31).

## Rationale for the Use of Nanoparticles

In the steady state, rapidly turning over immature DCs become tolerogenic and induce Tregs that maintain immune tolerance. In autoimmune diseases, instead, immature DCs become immunogenic and support pathogenic effector cells, with resulting predominant pathogenic T cells over the regulatory cells that should control them. The therapeutic objective, then, is to formulate NPs that can reset a dysregulated immune system back to normal and restore autoantigen specific Treg predominance. Since in some autoimmune diseases such as SLE, type 1 diabetes (T1D) and multiple sclerosis, specific autoantigen peptides have been identified, the goal is to induce antigen-specific Tregs. However, in diseases such as rheumatoid arthritis and inflammatory bowel disease where specific autoantigens are unclear, the goal is to target NPs to disease sites to switch immunogenic DCs to tolerogenic DCs and switch local macrophages from inflammatory to anti-inflammatory cells.

**Figure 2:**
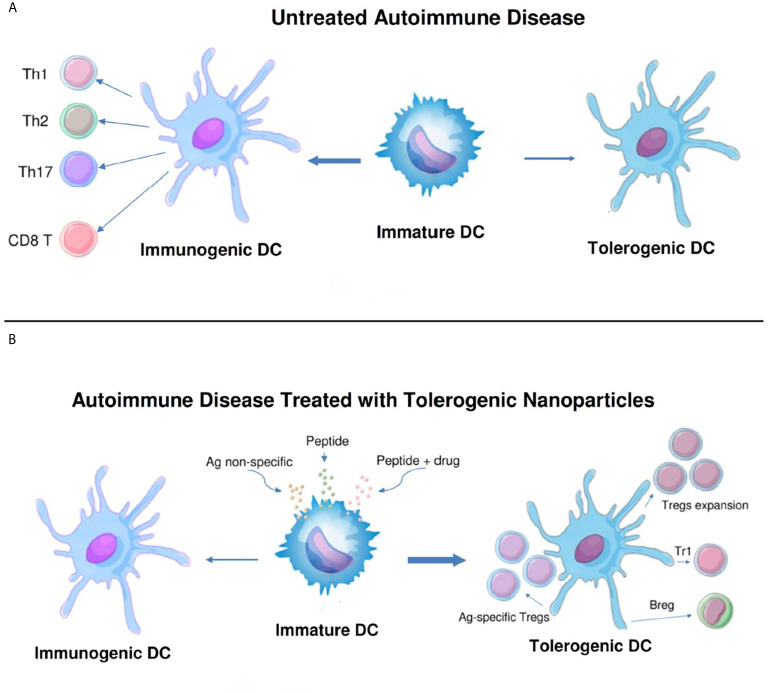
Nanoparticles targeted to antigen-presenting cells can switch immunogenic dendritic cells to tolerogenic. **(A)**. While immature dendritic cells (DCs) normally mature to tolerogenic in the steady state, in untreated autoimmune disease these cells can become immunogenic and induce pathogenic T cell effector cells (CD4^+^ Th1, Th2 and Th17, and CD8^+^ T cells). **(B)**. Different formulations of nanoparticles (antigen non-specific, peptide-containing, or peptide plus drug) have been designed to switch the maturation of DCs from immunogenic back to tolerogenic. These DCs expand one or more populations of regulatory cells (antigen-specific and non-specific CD4^+^ and CD8^+^ Tregs, Tr1 cells, and B regulatory cells) and reset the immune system to restore a predominance of regulatory cells over pathogenic effector cells.

**Figure 3:**
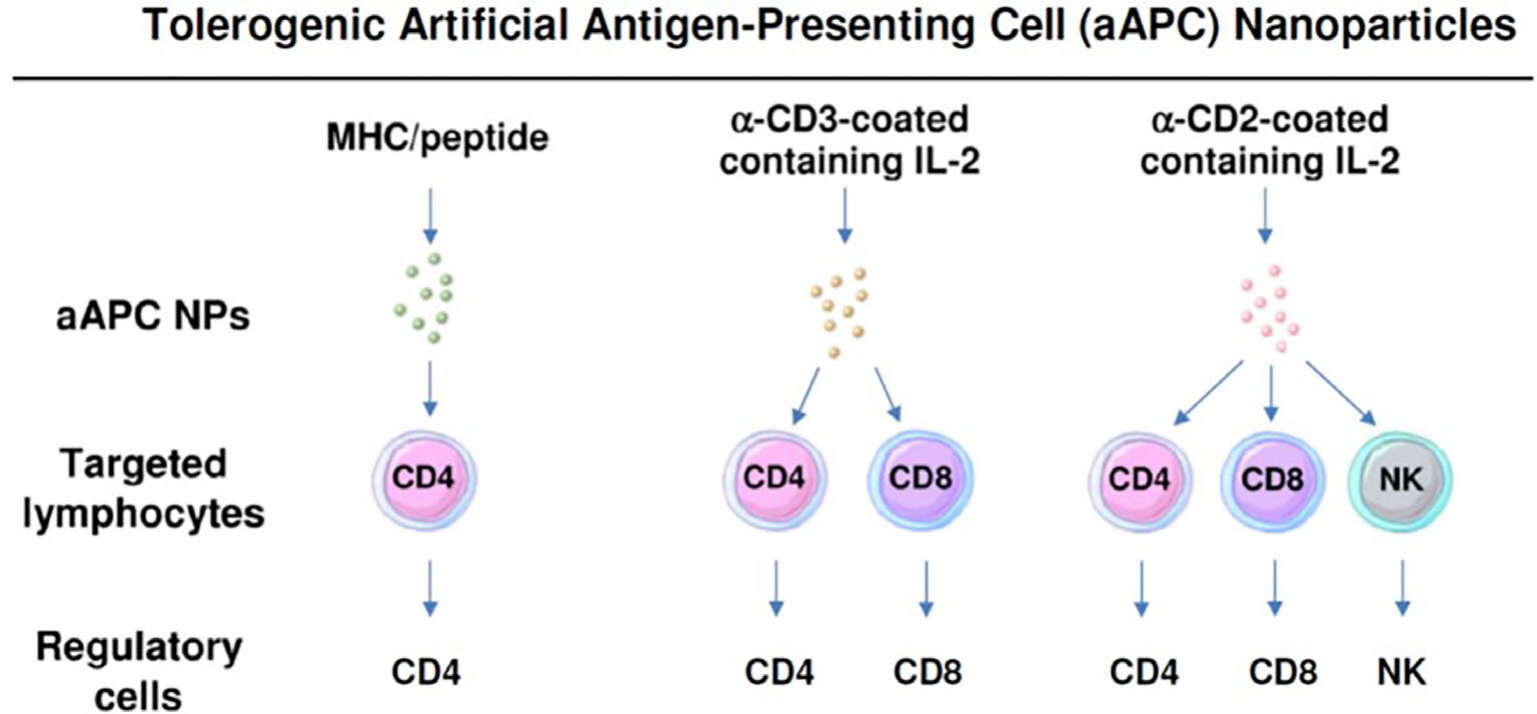
Nanoparticles can be formulated as tolerogenic artificial antigen-presenting cells that directly target specific lymphocyte subpopulations to become regulatory cells. Three examples are shown that induce one or more subsets of regulatory cells.

To restore Treg predominance, two approaches are possible: 1) NPs targeted to DCs or other antigen-presenting cells (APCs), to induce them to become tolerogenic, or 2) NPs targeted directly to T cells for the induction and expansion of Tregs. **Figures 2** and **3** summarizes these approaches. It has been established that CD4 Tregs require IL-2, TGF-β, and continuous T cell receptor stimulation for function and survival (7, 32, 33). Nanoparticles can provide these agents and, where possible, the antigen for the generation of antigen-specific Tregs. Also, although most investigators have focused on CD4 Tregs, CD8 Tregs have as well important tolerogenic roles (34, 35). In human SLE, like CD4 Tregs, CD8 Tregs can inhibit anti-DNA autoantibodies (36, 37). Therefore, attention should be given to inducing CD8 as well as CD4 Tregs.

## Nanoparticles That Generate Tolerance Through Modulation of Antigen Presenting Cells (APCs)

### Delivering Pharmacological Agents to Promote Tolerogenic APCs

The liver and the intestinal immune system are enriched in APCs with high tolerogenic potential (38, 39). It is well known that oral administration of protein antigens can result in non-responsiveness to those antigens. Oral tolerance can prevent certain autoimmune diseases in animals but, unfortunately, multiple attempts to translate that to human therapeutics has not been successful (39). Nanoparticles have been used to amplify tolerogenic effects (40). Repeated oral delivery of chitosan-DNA NPs can prevent antibodies blocking functional FVIII in mice with hemophilia A (41). Oral gene application using chitosan-DNA NPs induce transferable tolerance (42). Orally delivered nanoparticle-curcumin has been reported to ameliorate experimental colitis *via* modulation of gut microbiota and the induction of Tregs (43, 44). Curcumin is a hydrophobic polyphenol prepared from the root of the perennial herb *Curcuma longa*, a member of the ginger family. Curcumin possesses a wide variety of biological functions, such as anti-inflammatory, anti-cancer, antioxidant, antimicrobial, wound-healing and hypoglycemic activities. Curcumin inhibits cell signaling pathways that include nuclear factor k light-chain-enhancer of activated B cells (NF-κB), signal transducer and activator of transcription proteins (STAT)3, nuclear factor erythroid 2-related factor 2 (Nrf2), reactive oxygen species (ROS), cyclooxygenase (COX)-2, and phosphatidylinositol 3-kinase (PI3K) (43). Strong cell signaling through NF-kB and the PI3K/Akt/mTOR pathway generates inflammatory cells or T effector cells, respectively, while weaker signals induce anti-inflammatory cells or Tregs. It is likely that modulating signaling from strong to weaker contribute to the many effects of curcumin (44). A recent breakthrough in the development of NPs capable of delivering biologicals orally will be described below.

### Delivering Disease-Relevant Antigens to APCs Through Naturally Tolerogenic Mechanisms

In the steady state, a variety of APCs in the liver are in a tolerogenic state and maintain local and systemic immune tolerance to self and foreign antigens. These APCs include DCs, macrophage-like Kupffer cells (KCs), liver sinusoidal endothelial cells (LSECs), and hepatic stellate cells (HSCs). Even hepatocytes can express low levels of major histocompatibility complex (MHC)-I/MHC-II and co-stimulatory molecules that maintain tolerance (38). Investigators have taken advantage of NPs that accumulate in tolerogenic liver APCs to treat autoimmune diseases (45–47). One group has used PLGA NPs targeted to the liver to induce antigen-specific immune tolerance in a pulmonary allergen sensitization model (48). Others have used nanoparticle-based autoantigen delivery to Treg-inducing liver sinusoidal endothelial cells to control autoimmunity in mice (49).

Various approaches have been investigated that combine antigen delivery with a strong tolerogenic signal. The objective is to induce rapidly turning-over immature dendritic cells (DCs) to differentiate into tolerogenic APCs. In subjects with autoimmune diseases, immature DCs become immunogenic cells which perpetuate the disease. Here one must switch their differentiation of immunogenic cells to tolerogenic. One approach is to take advantage of the tolerogenic effects of clearing cells that died of apoptosis. Macrophages and immature APCs that phagocytose apoptotic cells produce TGF-β, which has tolerogenic effects (50–52). One group used the tolerogenic effects of apoptotic cells as a starting point for immunotherapy using the experimental allergic encephalomyelitis (EAE) mouse model of multiple sclerosis, characterized by T helper type 1 (Th1) and/or Th17 effector cells. The authors found that intravenous administration of peptides crosslinked to syngeneic splenic leukocytes safely and efficiently induced antigen-specific immune responses, and that the tolerance by apoptotic antigen-coupled leukocytes was induced by PD-L1+ and IL-10-producing splenic macrophages and maintained by Tregs (53). The same group then switched from antigen-labeled cells to antigen-bearing NPs. They observed that intravenous delivery of negatively charged PLGA NPs were taken up by splenic macrophages that express the scavenger receptor MARCO. These NPs prevented/treated EAE and T1D (54, 55), and the apoptotic effect of NPs carrying antigen taken up by phagocytic immature APCs led to the production of and TGF-β and IL-10. These cytokines matured the APCs into tolerogenic, with the ability to induce Tregs. Most recently, it has been shown that PLGA NPs carrying single or multiple peptides could induce CD4+Foxp3+ Tregs that suppressed CD4 and CD8 cells (56).

Macrophage recognition of phosphatidylserine, a component of the cell membrane, is another strong apoptotic signal that can increase tolerogenic IL-10 and TGF-β. Liposomes containing peptide antigen and phosphatidylserine were given to patients with T1D to determine their tolerogenic effects on DCs. These liposomes decreased the autologous T cell proliferation. However, likely because of variability of the DC responses, liposomes did not affect the profile of pro-inflammatory or anti-inflammatory cytokines released by the cells (57).

### Delivering Drug-Antigen Combinations to Drive Antigen Specific Tolerogenic Skewing

Nanoparticles that carry antigen-peptides and pharmacological agents have been studied for their capacity to generate Tregs. These agents attached to the surface or encapsulated in the PLGA NPs include TGF-β (58) and dexamethasone (59). Various immunomodulators have been used together with antigen-peptides to induce Tregs. Colloidal gold NPs have been engineered to deliver both a tolerogenic aryl hydrocarbon receptor (AHR) ligand and a proinsulin peptide to induce tolerogenic DCs that promote CD4+Foxp3+ Treg generation *in vivo* and prevent T1D in mice (60). These NPs induce monocyte-derived DCs to develop a tolerogenic phenotype by inhibiting NFkB signaling. The strength of cell signaling plays an important role in cell differentiation. The development of mature immunogenic DCs requires strong NFkB pathway signaling (61). By contrast, weaker NF-κB signaling is important in the establishment of immune tolerance, including both central tolerance and the peripheral function of Tregs (62). This AHR effect depends upon the induction of the suppressor of cell cytokine-2 (SOCS2) protein (60). These AHR-ligand containing NPs have been previously shown to induce Type 1 (Tr-1) Tregs and B regulatory cells (63). More recently this group has used nanoliposomes carrying an AHR ligand to treat EAE (64).

PLGA NPs containing antigen and an inhibitor of the PI3K/AKT/mTOR pathway have also been extensively studied for their tolerogenic effects. The PI3K pathway is the chief signaling pathway that T cells use to transmit antigen stimuli from the TCR to the nucleus (65). Similar to NFkB, strong TCR signals result in T effector cell differentiation, whereas weaker signals results in Treg differentiation (66). Rapamycin (rapa) inhibits signaling through mTOR. Although rapa has immunosuppressive effects, the combination of this agent and IL-2 promotes the induction of CD4+CD25+Foxp3+ cells (65). Rapa packaged in PLGA NPs has much stronger immunomodulatory properties than its soluble form (67). Nanoparticles containing antigen and rapamycin induce CD4+Foxp3+ Tregs and prevent EAE (67, 68). Many of the biological agents now in use for the treatment of human autoimmune diseases are antigen and can elicit antibodies that block their therapeutic effects. Tolerogenic polylactide NPs that block the production of these antibodies can have useful beneficial effects and are in clinical trials (69).

It is desirable to have antigen in the NP, yet antigen non-specific microparticles can also be useful. Blocking the positive co-stimulatory effects immunogenic DCs can be therapeutic. In T1D, three antisense oligonucleotides contained in microspheres were targeted to the primary transcripts of CD40, CD80 and CD86 co-stimulatory molecules. The result was attenuated T cell signaling that induced CD4+Foxp3+ Tregs which reversed hyperglycemia (70). In a lupus-like disease model resulting from a CD4 helper cell-driven chronic graft versus host disease, NPs induced CD4 and CD8 polyclonal Tregs that prevented the disease (31) Here the antigen source was non-self MHC peptides. Thus, with persistent endogenous antigen stimulation, polyclonal Tregs can have therapeutic effects.

## Nanoparticles With Direct Tolerogenic Effects on Lymphocyte Subsets

### Delivering Small-Molecule Drugs or miRNA to T Cells

Nanoparticles can have direct effects on T cells and B cells. NPs have been used to correct decreased T cell production of IL-2 and increased production of IL-17 in SLE (2). Calcium/calmodulin protein kinase IV has a role in both abnormalities. KN93, a small molecule inhibitor of this kinase, was encapsulated in a nanolipogel that was targeted to CD4+ cells. Previously, this group had reported that the soluble form of this inhibitor increased CD4+ Foxp3+ Tregs (71). Here the NPs markedly reduced murine EAE and SLE (72). T cells were not depleted, but Th17 cells were effectively blocked. In SLE lupus prone mice, targeted delivery of a CaMK4 inhibitor to podocytes preserved their ultrastructure, prevented immune complex deposition and crescent formation, and suppressed proteinuria. In animals exposed to adriamycin, podocyte-specific delivery of a CaMK4 inhibitor prevented and reversed podocyte injury and renal disease (73).

Aberrant DNA demethylation in T cells leads to T cell abnormalities in SLE and correlates with disease activity (74). 5-azacytidine, (5-azaC) a DNA methyltransferase inhibitor can correct these abnormalities. However, generalized hypomethylation can have many adverse side effects. Therefore, 5-azaC was packaged in liposomes that were targeted to either CD4 or CD8 cells. Each of these liposomes markedly improved nephritis in a mouse model of lupus. The mechanism of action on each T cell subset was different. The CD4-targeted liposomes increased Foxp3 expression, expanded CD4 Treg numbers and enhanced function. The CD8-targeted liposomes enhanced cytotoxicity of these cells and restrained the expansion of pathogenic TCR-αβ^+^CD4^–^CD8^–^ double-negative T cells. Importantly, systemic azaC delivery did not have these positive therapeutic effects (75). Thus, established disease could be reversed in a mouse model, underlining the importance of targeting NPs to specific cells.

In addition to T cells, liposome NPs have been used to target antigen directly to B cells. Antigenic liposomes displaying CD22 ligands induce antigen-specific B cell tolerance (76) and apoptosis (77).

Nanoparticles packaged with microRNA-125a (miR-125) have been reported to ameliorate a mouse model of lupus by restoring the balance between effector and Tregs. A miRNA is a small non-coding RNA molecule that functions in RNA silencing and post-transcriptional regulation of gene expression. miRNAs regulate approximately 90% of protein-coding genes (78). MiR-125 may have an important role in immune tolerance. One group reported that miR-125 is decreased in SLE patients (79). To repair this defect and increase stability of this RNA, miR-125 was packaged into ~150 nM NPs consisting of polyethylene glycol, PGLA, and poly (L-lysine). These NPs were endocytosed into activated T cells that became Tregs when cultured with TGF-β. Comparative *in vivo* studies in lupus mice with free miR-125 revealed that the NPs increased RNA concentration in the spleen and prevented splenomegaly and renal disease. This was accompanied by increased percentages of CD4 Tregs and decreased percentages of CD4 Th17 cells. Thus, in SLE, these NPs appear to have major effects on restoring normal immune regulation (80). However, miR-125 may have different properties in other diseases. In rheumatoid arthritis, levels of miR-125a are high and correlate with other inflammatory markers (81). In bacterial sepsis, high levels of this miRNA correlate with acute respiratory distress syndrome (82).

## Nanoparticles That Function as Tolerogenic Artificial Antigen-Presenting Cells (aAPCs) That Provide Activating, Costimulatory, and Cytokine Signals

Several groups have tried to substitute DCs or other APCs with NPs to make artificial antigen presenting cells (aAPCs). While previously immunogenic aAPCS had been formulated to enhance immunization (83), two approaches were undertaken to generate tolerogenic aAPCs. One provided both CD4+ and CD8+ cells the T cell receptor stimulation and cytokines to become Tregs. The other used NPs to present peptide-MHC complexes directly to T cells to induce CD8+ and CD4+ Tregs.

In 2011, it was shown that PLGA NPs coated with anti-CD4 antibodies and loaded with Leukemia Inhibitory Factor (LIF) induced mouse CD4+ cells to become CD4+ Foxp3+ Tregs (84). These NPs blocked the ability of IL-6 to induce CD4+ cells to become pro-inflammatory IL-17-producing cells. NPs encapsulated with LIF have used as neuroprotective in multiple sclerosis to repair myelin *in vivo (* 85, 86). This work was followed up in 2015 by loading CD4-targeted PLGA NPs with IL-2 and TGF-β, the cytokines that induce Foxp3 Tregs. These NPs induced mouse CD4+ cells to become Tregs that, unlike those induced with soluble IL-2 and TGF-β, were stable in the presence of IL-6. The percentage of nanoparticle-induced CD4 Tregs and their suppressive activity *in vitro* was much greater than those induced *ex vivo* by soluble IL-2 and TGF-β. Since CD4 Tregs need continuous IL-2 exposure to maintain Foxp3 expression (87), a single dose of NPs sustained Foxp3 expression for 10 days. By contrast, those CD4 cells stimulated with soluble IL-2 and TGF-β had completely lost Foxp3 expression by this time (13). At present, clinical trials are underway with low dose IL-2 to treat SLE. One has been completed: NCT 02084238. Ongoing trials include: NCT02955615, NCT03312335, NCT03451422, NCT03782636, and NCT02411253. While increases in Foxp3 quickly fall after each dose of IL-2, one might anticipate that NPs targeted to CD4+ cells that are loaded with this cytokine will sustain Foxp3 expression longer.

PLGA NPs targeted to both CD4 and CD8 cells and encapsulated with IL-2 and TGF-β have been used to prevent a lupus-like syndrome (chronic graft versus host disease) (31). In their studies with Tregs induced *ex-vivo*, this group had documented that the combination of CD4 and CD8 Tregs was more effective than CD4 Tregs alone in preventing this lupus-like syndrome (88). Their objective, therefore, was to expand CD4 and CD8 Tregs *in vivo.* To do so, they coated the NPs with both anti-CD2 and anti-CD4 antibodies. Anti-CD2 antibody was chosen since it had been reported that these antibodies can also target natural killer (NK) cells (89). This model was chosen because of its rapid read-out. It involves the transfer of mouse DBA/2 T cells into (C57BL/6 × DBA/2) F1 (BDF1) mice. Unlike most mouse strains, DBA/2 mice lack T cells that can kill CD8 cells and the ensuing graft versus host disease, therefore, is characterized by unopposed T cell help for antibody production. The result is a rapid onset of anti-DNA autoantibody production and a rapidly lethal immune complex-induced glomerulonephritis. In this model, the administration of these T cell and NK cell-targeted NPs containing IL-2 and TGF-β markedly suppressed disease.

In addition to mouse cells, tolerogenic aAPC NPs containing IL-2 and TGF-β have induced human CD4+ and CD8+ cells become Foxp3+ Tregs that were functional both *in vitro* and modulated systemic autoimmunity in humanized NOD/SCID immunodeficient mice. For the *in vitro* studies, the NPs were coated with anti-CD3 and anti-CD28 antibodies. For the *in vivo* studies, the NPs were anti-CD3 antibody-coated NPs containing IL-2 and TGF-β. After the transfer of human PBMC to the immunodeficient mice, treatment with aAPC NPs for three weeks resulted in increased CD4+ and CD8+ Foxp3+ cells that persisted until the termination of experiment. This was accompanied by increased survival of the human anti-mouse GVHD (90).

Another approach to use NPs as aAPCs is to present peptide-MHC complexes directly to T cells. In 2010 one group used NPs that carried peptide-MHC class I complexes to delete a subset of diabetogenic CD8+ cells in NOD mice. Although these NPs did restore blood sugar to normal levels in mice with new-onset diabetes, they unexpectedly expanded a subset of CD8+ cells that were autoregulatory cytotoxic cells that suppressed polyclonal autoimmune responses by killing autoantigen-loaded APCs in target tissue and draining lymph nodes (91). These workers then turned their attention to disease-relevant peptide-MHC class II complexes to expand therapeutic CD4 Tregs. They identified pMHC complexes that reversed diabetes, EAE and collagen arthritis in mice (92). The NPs targeted antigen-experienced pathogenic IFN-γ producing T helper 1 (Th1) cells and switched these cells into T regulatory type 1-like (Tr1-like) cells that produce predominantly anti-inflammatory IL-10. The Tr1 cells induced B cells to become IL-10-producing B regulatory cells. They documented ten pMHC class II complexes that had similar effects. Subsequently, they have identified complexes of non-organ peptides from mitochondria, nuclear or cytoplasmic proteins with MHCII have that induced therapeutic Tr1 cells in mouse models of liver diseases. These included primary biliary cirrhosis, primary sclerosing cholangitis and autoimmune hepatitis (93).

Another group engineered tolerogenic NPs co-coupling a myelin peptide-MHC complex, anti-Fas antibody, PD-L1-Fc and encapsulated with TGF-β These NPs decreased Th1, Th17, and Tc17 cells and increased Tregs. In EAE, mice that were treated early after disease onset responded well, but those treated with more advanced disease did less well (94). In addition to EAE, a study in skin transplantation with similar NPs co-coupling MHC class I dimers, CD47 and regulatory molecules showed that the NPs bound and induced apoptosis of CD8 cells, induced Tregs and improved transplant survival (95). Like the aAPC study described above, the work was conducted on mice with a C57/BL background. Since human autoimmune diseases occur in subjects with a much more diverse genetic background, obstacles remain for clinical translation as well as for manufacturing challenges.

## Nanoparticles That Induce Tolerogenic TGF-β-Dependent Regulatory NK Cells

Nanoparticles coated with anti-CD2 antibodies target NK cells as well as T cells. Studies were, therefore, undertaken to determine whether NK cells had a role in the protective effects of anti-CD2 antibody-coated NPs loaded with IL-2 and TGF-β in the lupus-like disease discussed above (96). Surprisingly, depletion of NK cells attenuated the NP-mediated increase in CD4+ and CD8+ Foxp3+ Tregs and exacerbated the resulting renal disease above the baseline of untreated mice (96).

Previously, anti-CD2 antibodies had been reported to induce NK cells to produce TGF-β (10, 97). This finding raised the possibility that TGF-β produced by NK cells could eliminate the need for this cytokine encapsulated in the anti-CD2 antibody- coated NPs. Additional studies were conducted with anti-CD2 antibody-coated NPs loaded with only IL-2 revealed that these NPs had equivalent protective effects on the renal disease as NPs containing both IL-2 and TGF-β. However, antagonizing TGF-β in the NP-treated mice by anti-TGF-β antibodies or with an Alk5 TGF-β signaling inhibitor abolished the protective effects. Thus, the protective effects of NPs loaded with only IL-2 were TGF-β-dependent.

Interestingly, NK cells harvested from the spleens of anti-CD2 antibody-coated NPs treated mice had equivalent protective effects on the lupus-like glomerulonephritis as the anti-CD2 antibody-coated NPs loaded with IL-2. Moreover, transfecting these NK cells with a silent RNA (sRNA) to inhibit TGF-β production completely abolished their protective effects. These studies provide evidence that the TGF-β produced by the NK cells may help in the maintenance and function of the CD4 and CD8 Tregs and, therefore, may play a major role in their protective effects (96).

## Nanoparticles Delivered Orally With Inherent Anti-Inflammatory and Tolerogenic Properties

Orally delivered NPs have been used to treat T1D in nonobese diabetic (NOD) mice. Oral polyethylene glycol (PEG)-PLGA loaded with insulin lowered glucose in T1D rodent models (98, 99). Orally delivered PLGA NPs with al-trans retinoic acid and TGF-β induced therapeutic Tregs in T1D (increased PD-1 and CTLA4 but not Foxp3) (100). However, the oral bioavailability of these NPs is only 1-2% because of intestinal degradation (101).

Recently, it has been reported that NP polymerization of ursodeoxycholic acid (pUDCA), a bile acid with well-known anti-inflammatory and immunomodulatory effects, markedly enhanced its therapeutic properties. In addition, pUDCA NPs had the capability to deliver insulin orally without intestinal degradation. These NPs were rapidly absorbed intact and taken up by monocytes and intestinal macrophages that highly express bile acid TGR5 receptors. This interaction results in their differentiation to M2 anti-inflammatory macrophages, an effect which had important therapeutic consequences. Two different mouse models of Type 1 diabetes were successfully treated with pUDCA NPs. Cyclophosphamide-induced diabetes was prevented with pUDCA NPs containing rapamycin. Treatment of hyperglycemic NOD mice with PUDCA NPs containing insulin lowered blood glucose, reversed inflammation, and increased survival. In both models the ratio of cytotoxic CD8 cells and CD4 Tregs in draining lymph nodes was reversed, a finding suggesting that immunogenic dendritic cells had been switched to tolerogenic. Thus, pUDCA NPs appear to be a first in class orally ingestible carrier with remarkable therapeutic properties applicable to a wide variety of immune-mediated inflammatory diseases (102).

## Discussion and Concluding Remarks

We have reviewed various approaches that use NPs to generate and expand Tregs by targeting APCs or directly targeting T cells and these approaches are summarized in **Table 1**. To induce and expand therapeutic polyclonal Tregs, NPs can be targeted to the large numbers of tolerogenic APCs present in the liver and in the intestinal immune system. Antigen-specific Tregs can be induced by including peptide antigens carried by the NPs. In autoimmune diseases approaches are directed to switch the differentiation of rapidly turning-over immature dendritic cells from immunogenic to tolerogenic. These include peptide-loaded NPs formulated to mimic the tolerogenic effects of particle apoptosis. A pharmacologic agent can be attached to or encapsulated in these NPs to enhance their tolerogenic properties. Alternatively, tolerogenic NPs can be formulated that directly target T cells or NK cells. NPs coating with peptide/MHC complexes target Th1 T cells and can switch them to become Treg1 cells in MHC compatible subjects. NPs coated with anti-CD2 or anti-CD3 antibodies can act as artificial APCs that target CD4 and CD8 cells that provide the T cell receptor stimulation, IL-2 and TGF-β that induce and/or expand polyclonal Tregs. These NPs have the potential to repair defects in IL-2 and/or TGF-β production associated with SLE and other autoimmune diseases and, thus, normalize Treg function. Since these anti-CD2 and anti-CD3 antibody-coated NPs have the additional property to induce their targeted lymphocytes to provide TGF-β in the local environment. These NPs therefore, contain only IL-2 (96). Because of the pleotropic activities of TGF-β, the possible adverse side effects of NPs containing TGF-β can be avoided. As indicated above, coating the NPs with anti-CD2 antibodies has recently been reported to induce NK cells to produce the TGF-β needed for the maintenance of Tregs.

**Table 1:** Different approaches employing nanoparticles therapies for tolerance induction.

Tolerogenic action through modulation of antigen-presenting cells			
Category	NP Description	Mechanism	References
Delivery of pharmacological agents to promote tolerogenic APCs	Multiple polymer- (PLGA) or lipid-based (liposome) NP formulations encapsulating immunomodulatory agents such as rapamycin, dexamethasone, vitamin D3 and curcumin	Induction of tolerogenic dendritic cell phenotype that can promote tolerance through a variety of mechanisms including Treg expansion and anti-inflammatory cytokine production. No antigen-specificity	40, 43, 44, 70
Delivery of disease-relevant antigen to APCs through naturally tolerogenic mechanisms	PLGA or chitosan NPs with encapsulated antigen	Oral delivery → Oral Tolerance	41, 42, 98–101
	Antigen-loaded pUDCA NPs (additional immunosuppressive property of polymer material)		102
	Antigen-loaded NPs designed to display signatures of apoptotic cells to exterior; examples include surface-bound phosphatidylserine and negative surface charge to promote internalization by MARCO receptor	Mimicry of apoptotic cells/bodies	53–57
	Antigen-loaded PLGA coated with ligands for mannose/scavenger receptors on LSEC	Targeting of naturally tolerogenic environments (liver sinusoidal endothelial cells, LSEC)	48
	Polymer-coated iron oxide nanocrystals or quantum dots with conjugated peptide antigen		49
Delivery of drug-antigen combination to APCs	PLGA NPs with co-encapsulated rapamycin and antigen or rapamycin only (delivered with free antigen)	Antigen delivery to APCs which are skewed tolerogenic by codelivery of immunomodulatory agents.	15, 67–69
	Gold NPs with conjugated peptide antigen and tolerogenic aryl hydrocarbon receptor agonist (later work with liposomes)		60, 63, 64
**Direct tolerogenic action on lymphocyte subsets**			
**Category**	**NP Description**	**Mechanism**	**References**
Delivery of small molecules to T cells	Nanolipogel system encapsulating CaMK4 inhibitor, KN93	Selective inhibition of CaMK4 in targeted CD4 T cells blocks Th17 differentiation	72, 73
	Nanolipogel system encapsulating DNA methyltransferase inhibitor, 5-aqzacytidine	Targeted demethylation leads to expansion and enhanced function of Tregs (CD4) cells and restrains expansion of pathogenic double-negative T cells (CD8)	75
Delivery of miRNA to T cells	Pegylated PLGA-b-poly(l-lysine) NP encapsulating miR-125a	Corrects imbalance of effector/regulatory T cells present in model of SLE	80
Delivery of cytokines to T cells	PLGA NPs encapsulating Leukemia Inhibitory Factor	Targeted delivery to CD4 T cells blocks IL-6 induced Th17 differentiation and favors upregulation of Tregs	84–86
	CD4/8-targeted PLGA NPs encapsulating TGF-β and IL-2	Paracrine delivery of cytokines promotes the induction and sustained expansion of CD4/8 Tregs with stable Foxp3 expression	13, 31, 90
	CD2-targeted PLGA NPs encapsulating TGF-β and IL-2	Targeted delivery of IL-2 to NK cells *via* anti-CD2 promotes expansion and upregulation of native TGF-β production	96
Peptide-MHC presentation to T cell receptors	pMHC complexes bound to surface of metal-oxide NPs	pMHC signal in the absence of costimulation promotes differentiation of IL-10 producing Tr1 cells and triggers deletion of pathogenic effector populations	91–93
Antigen delivery to B cells	Liposomes displaying both antigen and glycan ligands of CD22	Antigen exposure in the presence of CD22 engagement initiates tolerogenic programming that promotes antigen specific B cell tolerance as measured by decreased autoantibody formation	76, 77
Combination of multiple approaches	PLGA NPs decorated with pMHC, CD47, and multiple regulatory molecules with encapsulated TGFβ	Inhibition of T cell proliferation with selective decreases in effector Th1/Th17. Upregulation of regulatory T cells. Increased TGF-β and IL-10 in CNS and spleen.	94, 95

There are significant challenges to be confronted in developing NN-based therapies for autoimmune diseases. First, the translation of laboratory formulations of therapeutic NPs up to large scale clinical grade numbers will be formidable (103). There are manufacturing challenges in standardization and quality control of large batches of NPs. Secondly, not only autoimmune diseases diverse in type, but the individual presentation of a given disease can vary considerably. The therapeutic effects can vary between the initial time of onset and the chronic phase of the disease. We believe the optimal time to treat these diseases will be early before organ damage occurs. We are also optimistic that NP treatment of highly susceptible subjects before the onset of clinical disease may be beneficial. For example, treatment of rheumatoid arthritis early with tumor necrosis factor antagonists had the best likelihood of achieving remission (104). Thirdly, the dose, timing and frequency of administration of the therapeutic NP must be carefully evaluated. Fourthly, in achieving the objective to induce antigen-specific Tregs, the causal peptide can differ in that patients affected. Finally, in clinical trials the concurrent use of other immunosuppressive drugs can greatly influence the therapeutic outcome.

Clinical trials using tolerogenic nanoparticle formulations have begun. The first indication has been to prevent the emergence of antibodies to biological agents that can interfere with their beneficial effects. Human proof-of concept for the mitigation of anti-drug antibodies has been demonstrated in a phase II study in patients with refractory gout with NPs that are that loaded with pegadricase, a pegylated formulation of uricase, an enzyme that breaks down uric acid. Since pegadricase is strongly immunogenic, the NPs also contain rapamycin which converts strong immunogenic signals mediated by the PI3K/Akt/mTOR pathway to weaker tolerogenic signals (69). In addition, clinical trials using low dose IL-2 to repair and enhance Treg function are in progress for the treatment SLE and other autoimmune diseases. In one of these studies patients with SLE and other chronic immune-mediated diseases were treated with intermittent doses of low dose IL-2 for 6 months with persistent increases in CD4 Tregs and clinical improvement of disease activity and severity (105).

Although the results with low dose IL-2 have been encouraging, it is likely that NPs directly targeted to T cells which are able to provide them the stimulation and small amounts of both IL-2 and TGF-β in the local environment for them to become Tregs can have even more beneficial therapeutic effects with additional safety. The judicious use of these NPs can possibly achieve long-term remission and, ultimately, prevent SLE and other chronic immune-mediated inflammatory diseases in highly susceptible individuals.

## Author Contributions

DAH wrote the manuscript. ALC and SB edited the manuscript, made direct intellectual contributions, and prepared the Table and Figures. All authors contributed to the work and approved it for publication.

## Funding

Supported in part by the NIH grants HD097531 and AI154935 to ALC, and GM007205 to SB.

## Conflict of Interest

DAH is the Founder of General Nanotherapeutics, LLC and has a financial interest in the Company.

The remaining authors declare that the research was conducted in the absence of any commercial or financial relationships that could be construed as a potential conflict of interest.
